# The gut-lung axis in influenza A: the role of gut microbiota in immune balance

**DOI:** 10.3389/fimmu.2023.1147724

**Published:** 2023-10-20

**Authors:** Guosen Ou, Huachong Xu, Jialin Wu, Shiqi Wang, Yaokang Chen, Li Deng, Xiaoyin Chen

**Affiliations:** School of Traditional Chinese Medicine, Jinan University, Guangzhou, China

**Keywords:** gut-lung axis, gut microbiota, influenza A, immunity, herbal medicine

## Abstract

Influenza A, the most common subtype, induces 3 to 5 million severe infections and 250,000 to 500,000 deaths each year. Vaccination is traditionally considered to be the best way to prevent influenza A. Yet because the Influenza A virus (IAV) is highly susceptible to antigenic drift and Antigenic shift, and because of the lag in vaccine production, this poses a significant challenge to vaccine effectiveness. Additionally, much information about the resistance of antiviral drugs, such as Oseltamivir and Baloxavir, has been reported. Therefore, the search for alternative therapies in the treatment of influenza is warranted. Recent studies have found that regulating the gut microbiota (GM) can promote the immune effects of anti-IAV via the gut-lung axis. This includes promoting IAV clearance in the early stages of infection and reducing inflammatory damage in the later stages. In this review, we first review the specific alterations in GM observed in human as well as animal models regarding IAV infection. Then we analyzed the effect of GM on host immunity against IAV, including innate immunity and subsequent adaptive immunity. Finally, our study also summarizes the effects of therapies using probiotics, prebiotics, or herbal medicine in influenza A on intestinal microecological composition and their immunomodulatory effects against IAV.

## 1 Introduction

**Figure 1:**
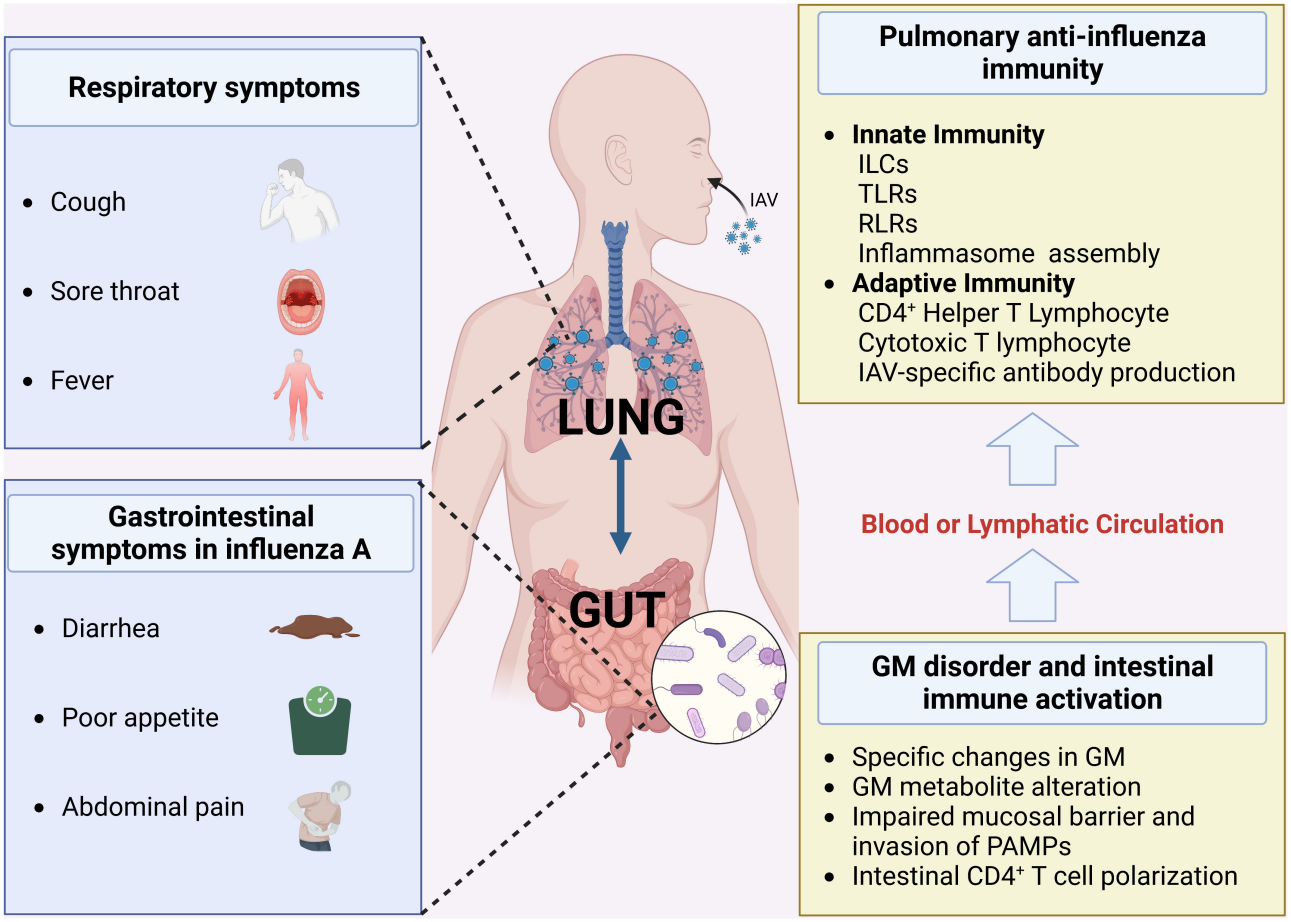
Gut-lung symptoms associated with influenza A and the mechanisms (Created with BioRender.com) (GM, gut microbiota; RLRs, RIG-I-like receptors; TLRs, Toll-like receptors; ILCs, Innate lymphoid cells).

The gut microbiota (GM), which includes bacteria, fungi, and viruses, contains more than 100 trillion microorganisms (1). Bacteria are the main component of it, and Firmicutes and Bacteroidetes phyla are predominant, fungi make up about 0.1% of it, and viruses contain even less (2). Although anatomically they are not directly related to the lungs when the GM is transformed into a pathogenic phenotype, or as a result, an immune imbalance in the intestine, it is a causative factor for many diseases (3). Recently, more and more researchers are focusing on the association between GM and lung diseases, such as chronic obstructive pulmonary disease, asthma, pneumonia, influenza, and COVID-19 (4–6). In particular, the immune regulation of GM and its metabolites on host defense against influenza A virus (IAV) involves almost the whole pathophysiological process of infection, including innate immunity, adaptive immunity. In the process, the relevance of mucosal immunity at various sites provides a pathway for IAV to affect the physiological functions of the intestine, while IAV does not colonize the intestine and exert direct pathological effects (7). For example, even though IAV is not present in the intestine, it can cause interferon-stimulated genes (ISGs) in the gut to express molecules with antiviral effects, which can affect the composition of the GM (8). It has also been found that IAV infection can affect the composition of the GM through the migration of Th17 cells from the lung to the intestine, or upregulation of intestinal ILC1s and ILC2s in the early stages of infection (9, 10). On the other hand, more studies have found that the process of host immunity against IAV, which occurs mainly in the lungs, is extensively regulated by the GM and its metabolites. This has been corroborated in germ-free animal models or antibiotic intervention models (11) (**Figure 1**). Therapies to regulate GM, such as probiotics, prebiotics, and herbal medicine, have a positive effect on the treatment of influenza A. In this review, we first review the specific alterations in GM observed in human as well as animal models regarding IAV infection. Then we analyzed the effect of GM on host immunity against IAV, including innate immunity and subsequent adaptive immunity. Finally, our study also summarizes the effects of therapies using probiotics, prebiotics, or herbal medicine in influenza A on intestinal microecological composition and their immunomodulatory effects against IAV.

## 2 The change of GM components in IAV infection

Although IAV ssRNA is not detected in the stool of influenza A patients, gastrointestinal symptoms such as diarrhea and loss of appetite are particularly common among them, especially in children (12). GM disorder is an important cause of these symptoms, especially the abnormal proliferation of *Escherichia_coli*, a conditionally pathogenic bacterium in the intestine, which is thought to be a common cause of vomiting and diarrhea (9).

IAV infection significantly up-regulated the Shannon diversity index and Chao1 index of the GM compared to the control group (13). On the analysis of difference GM, our previous study found that the abundance of *Escherichia_coli*, *Helicobacter_hepaticus*, and *Clostridium_perfringens* increased in the cecum feces of A/Fort Monmouth/1/1947 (H1N1) (FM1)-infected mice, while the abundance of the probiotics *Desulfovibrio_C21_c20* and *Lactobacillus_salivarius* decreased (14). For mice infected with others types of the H1N1 virus, A/Puerto Rico/1934 (H1N1) (PR8), the proportion of *Segmented filamentous bacteria* (SFB) and *Lactococcu*s in their stools increased (12). The effect of IAV on GM was also observed in a pig model, where the diversity of the intestinal flora was increased but the longitudinal development of *Lactobacillaceae* was disturbed compared to the control group, resulting in a significant decrease in the abundance of *Streptococcaceae (* 15). Silan Gu and his team sequenced the GM of 24 H1N1 patients in Zhejiang, China, and found a significant decreases in *Enterococcus*, *Prevotella*, *Finegoldia*, *Peptoniphilus*, and butyric acid-producing intestinal flora, including *Blautia*, *Agathobacter*, and *Anaerostipes (* 16). Franc¸ois Trottein’s team also observed a decrease in the abundance of short chain fatty acid (SCFAs)-producing bacteria in the stool of mice 7 days after IAV infection, including *Lachnospira-ceae*, *Lactobacillaceae*, and *Bifidobacteriaceae* (17). Subsequently, the abundance of these groups returned to normal 14 days after infection (17). Similar results were seen in the IAV-infected pig model (15). Studies on fungal and viral alterations in the intestine caused by IAV infection are currently lacking. Notably, several recent studies have identified intestinal fungal and viral alterations in COVID-19 patients. Tao Zuo et al. found an increased proportion of *C. albicans* in the stools of COVID-19 patients, and some of the patients also found *Asper-gillus* pathogens, a fungus that colonizes the respiratory tract and causes a variety of respiratory symptoms (18). In terms of viral alterations in the gut, another study from their team found a decrease in *Pepper mild mottle virus* and multiple bacteriophage lineages in the stool of COVID-19 patients and an enrichment of environment-derived eukaryotic DNA viruses (19). The above evidence reflects that respiratory virus infection may be accompanied by changes in enteroviruses and fungi. However, considering the differences in pathogenesis and pathophysiological changes among different respiratory viruses, the view still needs to be treated with caution and further studies.

Overall, the GM changes caused by IAV infection were mainly characterized by decreased levels of probiotic bacteria such as butyric acid-producing bacteria, meanwhile increasing levels of conditionally pathogenic bacteria. However, the vast majority of studies have focused only on changes in the bacteria at a certain timepoint after influenza infection (mostly at days 7-14). Studies on the dynamic changes of the GM during influenza are still lacking.

The disruption of GM and its metabolites is not merely a consequence of IAV infection, but an essential factor in the subsequent impact on antiviral immunity. Pathogen clearance after IAV infection is modulated by the GM. Our previous study also found that viral load in Bronchoalveolar Lavage Fluid (BALF) was significantly elevated in mice with disrupted GM caused by pretreatment with neomycin after infection with IAV (20). Furthermore, the composition of the GM has been correlated with the regulation of inflammatory damage following IAV infection and the development of secondary bacterial infections.

## 3 Gut microbiota affects innate immunity to influenza A

### 3.1 GM affects the activation of ILCs in influenza A

**Figure 2:**
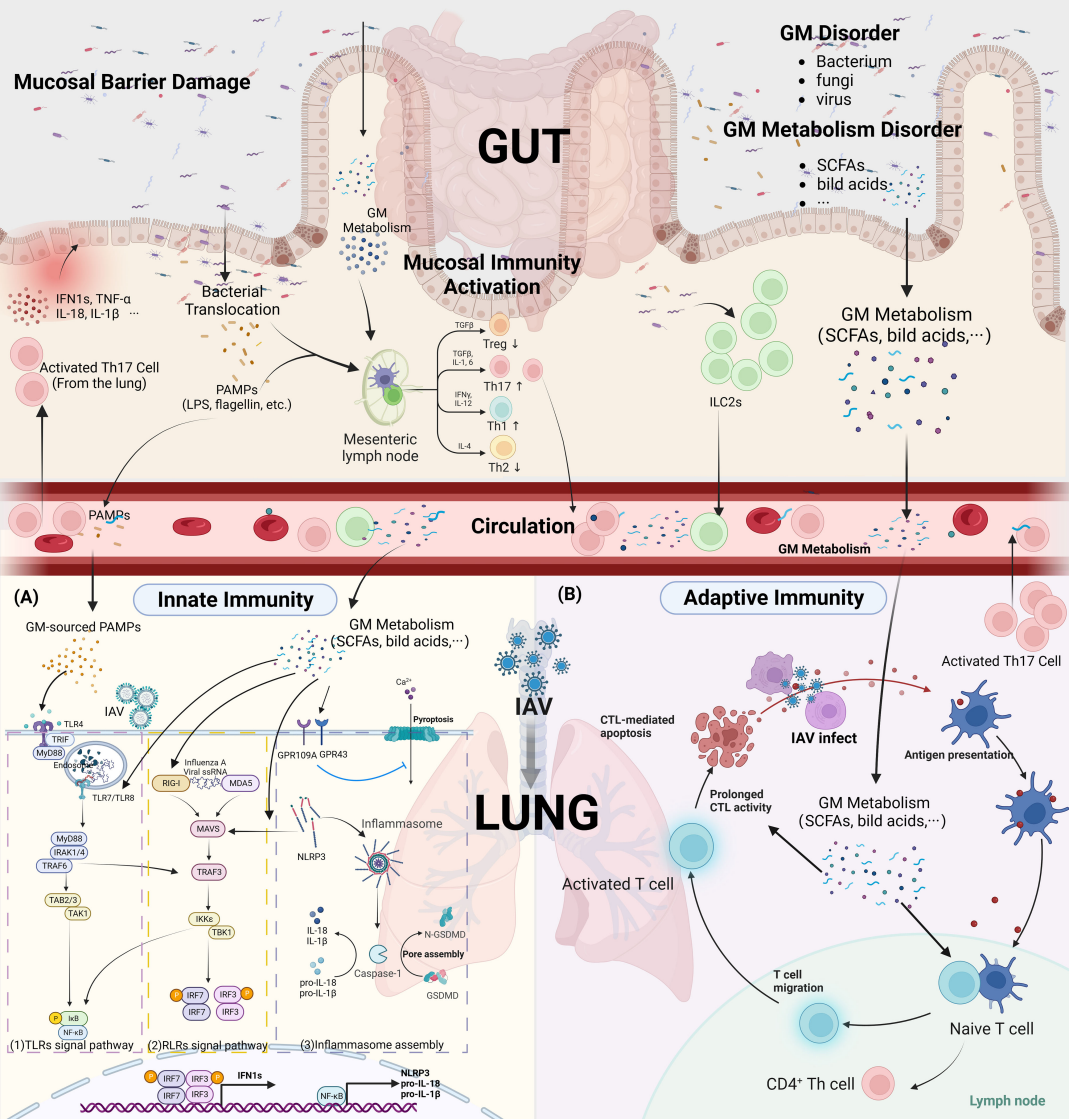
GM affect innate and adaptive immunity in IAV infection. (Created with BioRender.com) **(A)** Innate Immunity (1). TLRs Signal pathway: GM-sourced PAMPs that reach the lungs via the circulation (i.e. LPS, flagellin) are recognized by TLRs, and IAV ssRNA that invade into the endosomes are recognized primarily by TLR7/8. GM metabolites upregulate TLRs among endosomes and enhances innate immunity to the initial phase of influenza A. TLRs that recognized PRRs were able to recruit activated MyD88. The classical NF-κB pathway is the activation of TRAF6 by MyD88 and IRAK-4, followed by the activation of TAK by TRAF6 and the degradation of I κB bound to NF-κB. Subsequently, NF-κB molecules enter the nucleus to drive transcription of pro-inflammatory factors such as pro-IL-1β, pro-IL-18. In addition, MyD88 and IRAK-4 also activate TRAF6, which leads to the formation of homodimers of IRF3, IRF7 by activation of TBK1, respectively, and into the nucleus to drive transcription of type 1 IFNs. (2). RLRs Signal pathway: GM metabolites upregulate RIG-I protein expression in lung innate immune cells and also activates MAVS in an NLRP3-dependent manner. The IAV ssRNA that invade the cytoplasm are recognized by RLRs (RIG-I AND MDA-5) and then act on the mitochondrial MAVS to form the MAVS signalosome, which phosphorylates the TBK1/IKK complex. TBK1 then drives IRF3, IRF7 to form a homodimers complex that enters the nucleus to drive transcriptional production of type 1 IFNs. (3). Inflammasome assembly: Activation of the NF-κB pathway provides the basic material for the assembly of inflammasome (NLRP3, PRO-IL-1β, PRO-IL-18), which are then activated by the combined stimulation of ion flow disturbances in the cell membrane caused by viral invasion, as well as by RLRs signal. It induces the release of pro-inflammatory factors IL-1β, IL-18 and the assembly of GSDMD pores in the cell membrane, causing pyroptosis. GM metabolites (SCFAs) can inhibit the pyroptosis process triggered by Ca^2+^ inward flow via activation of GPR43, GPR109A on the cell surface. **(B)** Adaptive Immunity: GM metabolites facilitate the migration of DCs cells to the draining lymph nodes, thus contributing to the antigen presentation process. Altered the differentiation direction of CD4^+^ T cells and elevated the cytotoxic effect of CD8^+^ T cells.(IKK, NF-κB p65 inhibitor protein kinase; ILCs, Innate lymphoid cells; IRAK-4, IL-1R-associatedkinases 4; IRF3/7, IFN-regulatory factor 3/7; IκB, inhibitor of NF-κB; LPS, lipopolysaccharide; LRRs, leucine-rich repeats; MAVS, mitochondrial antiviral signaling; MyD88, myeloid differentiation primary response protein 88; NACHT, central nucleotide-binding and oligomerization domain; NF-κB, nuclear factor-κB; NLRP3, NOD-, LRR-, and Pyrin Domain-Containing Protein 3; RLRs, RIG-I-like receptors; TAK, TGFβ-activated kinase; TANK1, TRAF associated NF-ĸB activator; TLRs, Toll-like receptors; TRADD, TNFR associated death domain; TRAF3/6, TNF receptor-associated factor 3/6; TRAIL, Tumor necrosis factor-related apoptosis inducing ligand; GSDMD, gasdermin D; LPS, lipopolysaccharide).

Although ILCs has traditionally been considered to be a type of tissue-resident cell, some evidence in recent years suggests that they appear to be one of the communication pathways of the gut-lung axis, especially the ILC2s. ILC2s are the main ILCs in the lung, accounting for approximately 85-90% of the total pool of ILCs (21).Respiratory ILC2s exert the role traditionally thought of as antiparasitic infections (22), but they are also involved in antiviral immunity in the lungs. Silver JS et al. found that IAV infection induces transformation of ILC2s into ILC1s in the lung of mice to promote antiviral effects, which is mediated by the local production of pro-inflammatory cytokines (IL-12, IL-18, and IL-33) during viral infection (21). In a direct migratory complementary manner, ILC2s from the intestine can affect the function of the pulmonary local ILC2s. Huang and colleagues confirmed that ILC2s in mesenteric lymph nodes can migrate through the blood circulation and can be locally recruited to lung (22). This process is mediated by IL-25 in the lamina propria of the intestinal mucosa (23). In a manner that affects the migration of intestinal ILCs to the lungs, or directly via the blood or lymphatic circulation, GM and their metabolites are involved in the regulation of the activation of ILCs in the lungs. SCFAs supplementation suppressed the increased number of ILC2s in the lungs via the FFAR2-related pathway (24). Similarly, Ya-Jen Chang and colleagues found that butyric acid can limit the proliferation of ILC2s in the lung by inhibiting histone deacetylase (HDAC) (25). These seems to suggest that supplementation with SCFAs or SCFAs producing probiotics early in IAV infection is disadvantageous to the clearance of the pathogen. In addition, ILCs also can migrate to the intestine after being activated in the lungs. IL-33, a product in the lung after IAV infection, can contribute to the migration of CCR2^+^ILC2s characteristic of the lung to the intestine and affect the homeostasis of intestinal mucosal immunity (26). In conclusion, the migration of ILCs seems to be involved in the immune disruption of the gut-lung axis of influenza A (**Figure 2**). However, further in-depth studies on the GM components or its metabolites in the interaction with ILCs in influenza are currently awaited.

### 3.2 GM affects the TLRs pathway of IAV recognition

Existing studies found that TLR3, TLR4, TLR5, TLR7, and TLR8 are involved in the recognition of PAMPs during IAV infection (27). TLR3, TLR7, TLR8 are all distributed on the endosomal membrane (28). The ssRNA of influenza virus is recognized by TLR7 and TLR8, and the dsRNA produced during viral replication is recognized by TLR3. TLR4, and TLR5 are not involved in the recognition of viral nucleic acids, however they recognize viral proteins and GM related antigens caused by viral infection, such as LPS (TLR4 ligand) and flagellin (TLR5 ligand). The TLRs-related anti-IAV pathway is widely regulated by the GM. In our previous study, we found that disruption of GM by antibiotic application significantly downregulated TLR7 signaling pathway-related proteins, especially TLR7 and NF-κB, in FM1-infected mice, and also inhibited the secretion of downstream pro-inflammatory factors IFN-γ and IL-17, while TLR7 signaling pathway was restored by oral administration of the probiotic *Bifidobacterium (* 29). Similarly, a study by Wei Chen and his colleagues found that the expression of TLR7, MyD88, TRAF6 and TNF-α was significantly increased in mice orally administered *B. breve CCFM1026* after FM1 infection (30). Pectin oligosaccharides, a prebiotic, benefited the growth of *Lactobacillus, Prevotella, Rilenellaceae*, and *Lachanospiraceae groups* in a Poly I:C (TLR3 agonist) simulated mouse model of viral infection. This also increased plasma levels of IgG and IgA and reduced the rise of inflammatory factors in the lungs (31). In addition to those located in the respiratory tract, recent studies have found that some PRRs located in the innate immune cells of the gut, such as TLR4 and TLR5, are also involved in anti-IAV immunity through recognition of GM components. TLR5, which recognizes the flagellin protein of GM, has been shown to impair plasma cell production and antibody responses to inactivated IAV vaccines by knockdown (32). Mice with GM disorders caused by antibiotics have worse outcomes after IAV infection, but these can be improved by topical administration of LPS, a PAMPs recognized primarily by TLR4, to the colon (33). A recent study shows that Outer membrane-associated polysaccharide A produced by Bacteroidetes phylum in the intestine affects host immunity to IAV by regulating the expression of type 1 IFNs in the intestine and body circulation in a TLR4-dependent manner (34). In general, TLRs in innate immune cells located in the gut and respiratory tract, mainly TLR7, TLR4, TLR3, and TLR5, are involved in early host immunity against IAV (**Figure 2**). Moreover, these receptors are important intermediates for the anti-IAV effects of GM components.

### 3.3 GM affects the RLRs pathway of IAV recognition

RIG-I-like receptors (RLRs) is a class of PRRs located in the cytoplasm that specifically recognizes viral RNA, mainly RIG-I and MDA5, whose activation is a key signal to promote the secretion of type 1 IFNs. Growing evidence establishes the process of virus recognition by RLRs and GM interactions. Blockage of the RLRs pathway can cause pathological transformation of the GM (35, 36), moreover, activation of the RIG-I pathway promotes tissue repair and restores the integrity of the intestinal barrier (37). Available studies suggest that metabolites of the GM, mainly SCFAs, are involved in regulating the recognition of viruses by RLRs. It is not the microbiota of the lungs, but the gut that is the main source of SCFAs in the lungs, although many microbiotas located in the respiratory tract have been reported to produce SCFAs as well (38). A recent study found that external supplementation with butyrate increased RIG-I receptor expression thereby facilitating rhinovirus clearance (39). *In vitro* research showed that the antiviral effect exerted by acetate was transduced through the RIG-I signaling pathway, and a loss of protection against respiratory syncytial virus infection by acetate was observed on RIG-I knockdown A549 cells (40). In contrast, the addition of acetate promoted the upregulation of RIG-I, MAVS expression, and thus ISGs expression in A549 cells (40). Niu J and colleagues found that MAVS located on mitochondria, in addition to receiving activation by RLRs signaling, also receives acetate produced by the intestinal bacterium *Bifidobacterium pseudolongum NjM1* via NLRP3, leading to increased production of the key antiviral molecule type 1 IFNs (41). In conclusion, SCFAs-producing bacteria from the intestine can enhance the recognition of IAV by RLRs in lung innate immune cells through the release of SCFAs and exert anti-IAV effects through the expression of effector molecules IFN1s and IFN3s.

### 3.4 GM affects inflammasome assembly in influenza A

Pyroptosis caused by the assembly of inflammasome is an important method of PRRs signaling-mediated immune clearance in innate immunity. The lack of NLRP3 is unfavorable for the control of IAV in the early stages of infection (42). The assembly of inflammasome in the lung is regulated by the components of the GM. The metabolites of the GM, SCFAs, can induce the activation of inflammasome by promoting Ca^2+^ inward flow through binding to GPR 43 and GPR 109 A (43). On the contrary, after antibiotic treatment in elderly patients, metabolomic analysis revealed that abnormalities in the GM interfered with bile acid metabolism, causing inadequate production of serum secondary bile acids and resulting in elevated NLRP3 inflammasome signaling (44). In parallel, antibiotic treatment reduced the mRNA expression of IL-1β, IL -18 and NLRP3 in BALF in a mouse model (33). After GM dysregulation, gut-derived antigenic entering the circulatory system can cause activation of inflammasome in distant organs. Yun Zhang and colleagues found that young rats receiving GM from older individuals had significantly increased plasma levels of LPS and upregulated expression of NLRP3 inflammasome in their atrial tissue (45). Similarly, LPS of intestinal Gram-negative bacterial origin can also cause activation of inflammasome in the lung, leading to pyroptosis of alveolar macrophages, and increased inflammatory damage in lung tissue, in a model of acute lung injury (46). Moreover, inflammasome activation in the gut caused by GM disorders can also be associated with inflammation-related diseases in other organs, such as depression, anxiety, Alzheimer’s disease, IgA nephropathy, through the secretion of pro-inflammatory factors IL-1β and IL-18 into the circulatory system (47–49) (**Figure 2**). However, there is no research evidence to suggest an association between intestinal inflammasome activation and respiratory IAV infection.

## 4 Gut microbiota affects adaptive immunity to influenza A

### 4.1 GM affects CD4^+^ helper T lymphocyte activation in influenza A

The differentiation of CD4^+^ naïve T cells is regulated by the GM and its metabolites. Th17 is mainly located in the mucosa, especially in the intestine, where it mediates the balance between microbiota and the host immune system, preventing the invasion of pathogenic bacteria (**Figure 2**). An intestinal commensal bacterium, SFB, which has received much attention in recent years, induces the secretion of serum amyloid A proteins from the intestinal epithelium, thereby promoting the differentiation of naïve T cells to pro-inflammatory Th17 cells (50). Another intestinal bacterium, *Lactobacilli*, can promote intestinal IL-22 expression by participating in tryptophan metabolism and producing ligands for an aryl hydrocarbon receptor (AhR), which consequently affects Th17 differentiation (51). The fungus *C. albicans* in the intestine was also found to promote Th17 cell differentiation, and this *C. albicans*-specific Th17 cell can be activated by airborne fungi to mediate subsequent lung inflammation (52). However, in a recent study, *C. albicans*-mediated activation of Th17 cells was not beneficial for anti-IAV immunity (52). Treg cells are able to reduce immune damage by secreting IL-10, TGF-β, as well as suppressing the cytotoxic effects of adaptive CD8^+^ T cells during the waning phase of infection, while promoting the maturation of memory CD8^+^ T cells (53). *Clostridium* spp. induces TGF-β release and causes increased differentiation of colonic Treg cells (54). In contrast, *L. reuteri*, a key bacterium in the catabolism of tryptophan, inhibits the expansion of RORγt Treg cells by generating AhR ligands (55). Additionally, the promotion of Treg cell differentiation by SCFAs has been well reported. Both acetate and propionate promote the local accumulation of Treg by activating GRP43, and propionate also promotes Treg accumulation by inhibiting histone deacetylase (HDAC) (56, 57). Butyrate also promotes differentiation of Treg cells in an intronic enhancer CNS1 (conserved non-coding sequence 1)-dependent manner (56).

During the immune clearance phase in the early stages of IAV infection, differentiation of CD4^+^ helper T lymphocytes in the lung is characterized by increased differentiation of Th17 cells, accompanied by elevated IL-17 production (58). In addition to the target organs of IAV (respiratory tract), this process also occurs in the intestine. CCR9^+^ CD4^+^ T cells located in the lung migrate to the intestine, driven by C-C motif ligand 25 (CCL25), expressed in the intestinal mucosa epithelium (58). This process can cause disturbance of GM, disruption of intestinal barrier function (8), and subsequent polarization of Th/Treg cells in the mesenteric lymph nodes and migration back into the lung, regulating pulmonary immunity (59). Overall, in the pre-infection phase of IAV infection, the differentiation of CD4^+^ helper T lymphocytes shows a predominant proliferation of Th1 and Th17, which have a role in promoting immune clearance of pathogens. In our previous study, we found that the differentiation of CD4^+^ helper T lymphocytes in the spleen of mice after 5 days of IAV infection showed an increase in Th1/Th2 and Th17/Treg ratios (14, 60). However, If the GM is pre-empted by antibiotic intervention, a significant decrease in the number of CD4^+^ helper T lymphocytes was observed after IAV infection, accompanied by impaired clearance of IAV from the lungs (33).

In the later stages of infection, viral replication is controlled by the action of adaptive immunity but is followed by inflammatory tissue damage caused by a large number of pro-inflammatory factors. This bring about damage to the mucosal barrier, which is a trigger for co-infection with bacteria (61). GM composition helps to regulate the differentiation balance of CD4^+^ helper T lymphocytes to reduce excessive inflammatory damage in the late stages of IAV infection. Our previous studies showed that transplantation of GM treated with GeGen-QinLian Decoction, an herbal formula with antiviral effects, downregulated the Th1/Th2, Th17/Treg ratios in splenic CD4^+^ helper T lymphocytes of IAV-infected mice and also downregulated IL-6 and IL-17 A levels in mLNs and serum (14). In addition, SCFAs can promote Treg cell differentiation in the intestine by histone deacetylase (HDAC) at the Foxp3 locus (61), thereby modulating the inflammatory response and attenuating neutrophil-mediated tissue damage (16).

### 4.2 GM affects the function of CTL in influenza A

Cytotoxic T lymphocyte (CTL) is a key player in the role of viral clearance. Nude mice lacking thymus are unable to clear IAV from the respiratory tract due to the lack of mature T cells, thus leading to the persistence of infection (62). Disturbances in the GM interfere with the function of CD8^+^ CTL in viral infections. Oral antibiotic treatment in mice down-regulated the levels of specific CD8^+^ T CTL, thereby exacerbating the disease severity of multiple flavivirus infections (63). Comparably, GM disruption induced by oral neomycin decreases the immune response to IAV in the respiratory CTL as the gut is depleted of Gram-positive bacteria (33). Activation and maturation of CTL depend on local DCs in the respiratory mucosa to recognize and present IAV antigens, which then migrate to the corresponding draining lymph nodes. DCs carrying pathogen signals stimulate naive CD8^+^ T cells located in the lung draining lymph nodes to transform into CD8^+^ T cells with the ability to kill IAV-infected cells (64). Takeshi Ichinohe and colleagues found a significant reduction in the number of CD103^+^ DCs in the lungs and mLNs of antibiotic-treated mice and lower expression levels of CD86, CD80 and MHC II molecules on DCs (33). The CTL then migrate to the site of infection and exert cytotoxic effects. Yet, pre-administration of oral probiotic *Lactiplantibacillus plantarum 0111* increased the percentage of CD3^+^CD8^+^TNF-α^+^ T lymphocytes in the spleen of mice (65). In addition, the increased production of SCFAs induced by a high fiber diet increased the tissue homing and cytotoxic capacity of CD8^+^ T lymphocytes, as reflected by a decrease in the expression of the lymph node homing marker CD62L and a higher proportion of cells expressing CD107, a degranulation marker (66). In conclusion, GM and metabolites contribute to the antigen-presentation process of DCs cells and the subsequent cytotoxic effects induced in CTL cells (**Figure 2**).

### 4.3 GM affects IAV-specific antibody production

The best means of preventing influenza at present is via influenza vaccination before the epidemic period, which allows the body to produce specific antibodies. The GM is involved in regulating this process. Recently, a large number of clinical trials have supported the effectiveness of probiotic preparations in increasing the protective efficacy of influenza vaccines and significantly improving antibody titers in vaccinated individuals (67, 68). The production of IAV-specific antibodies is critical for IAV clearance. However, it was found through animal experiments that the use of antibiotics prior to vaccination not only reduced the titer of vaccine-induced antibodies, but also decreased the antigen-neutralizing ability of the antibodies produced (44, 69). Similar results were found in germ-free mice, and re-transplantation of the intestinal flora restored the protective efficacy of the vaccine (44, 70). Moreover, inadequate production of SCFAs by GM compromises the differentiation of B cells into pathogen-specific antibody-producing plasma cells (71).

## 5 Gut microbiota and bacterial co-infection secondary to influenza A

Respiratory viral-bacterial co-infections often lead to severe lower respiratory complications and result in a very high mortality rate. Among them, IAV combined with *S. pneumoniae* and *Haemophilus influenzae* are particularly common (72). Damage to the mucosal barrier caused by the highly pro-inflammatory state after IAV clearance is an important trigger for late influenza co-infection with bacteria (61, 73). The mechanism for regulating the components of the GM to prevent bacterial co-infection after influenza may be through inhibition of neutrophil exudation in the lungs and reduction of inflammatory damage. Mice fed a high-fiber diet or supplemented with butyrate down-regulated the expression of neutrophil chemokine CCL1 in the respiratory tract and reduced the number of neutrophils in BALF (66). As influenza causes a decline in the SCFAs-producing flora of the gut, the ability of alveolar macrophages to phagocytose pathogenic bacteria is also reduced, and this can be restored by supplementation with acetate (17). Pathogenic bacteria located in the gut are also a risk factor for bacterial co-infection. A large amount of research evidence suggests that SCFAs contribute to the repair of the intestinal mucosal barrier. During IAV infection in mice, supplementation with SCFAs reduced the entry of enteric pathogens, such as *FITC-dextran* and *Salmonella*, into the circulatory system, which was achieved by enhancing the mucosal barrier function in the intestine (8, 61). Similar findings were found in our previous research: decreases in the intestinal mucosal epithelial tight junction proteins (claudin-1, ZO-1, and Occludin) were found in IAV-infected mice, and their levels were restored after transplantation into the GM of mice receiving oral GeGen-QinLian decoction (14). In addition to the local mucosal barrier of the intestine, a recent study found that some probiotics can also enhance the epithelium of the lung. *Clostridium butyricum*-induced ω-3 fatty acid (18-HEPE) promotes lung tight junction protein expression through upregulation of IFN-λ (74). Overall, viral-bacterial co-infection occurs mainly in the late stages of IAV infection, while modulation of the GM and its metabolites, mainly SCFAs, prevents pathogenic bacteria from invading by reducing neutrophil exudation to attenuate lung inflammatory damage, repairing the mucosal barrier, and enhancing phagocytosis of pathogenic bacteria by macrophages.

## 6 Gut microbiota modulation influenza A virus infection

### 6.1 Probiotics and prebiotics

The gut is the largest immune organ. Regulation of GM has been shown to improve a wide range of inflammation-related diseases in multiple systems throughout the body such as Inflammatory bowel disease, cancer, Alzheimer’s disease, and depression (75–77). A meta-analysis of eight clinical trials involving 726 patients showed that probiotic administration reduced the risk of respiratory viral infections, among which the commonly used probiotics were *Lactobacillus, Bifidobacterium*, and *Lactococcus (* 78). Existing studies have found that probiotic formulations, prebiotics, probiotics, or herbal medicine, modulate both innate and adaptive immunity, thus acting to promote pathogen clearance and reduce inflammatory damage.

In particular, in terms of pathogen clearance, M Kawase et al. found that oral pretreatment with *Lyophilized Lactobacillus rhamnosus GG* and *Lactobacillus gasseri TMC0356* significantly reduced the viral load in the lungs of mice 6 days after PR8 infection (79). Additionally, oral feeding of *boiled Lactobacillus plantarum 06CC2*, *Lactobacillus plantarum DK119*, *heat-killed b240*, and *L. paracasei* alone also reduced viral load in BALF after IAV infection (80–83). Oral administration of *Lactobacillus gasseri SBT2055* reduces viral load in the lungs of PR8-infected mice 5 days after infection and also upregulates the expression of the antiviral genes Mx1 and Oas1a (84). The reduction of viral load in the lungs by probiotic treatment may be linked to its promotion of innate as well as adaptive immunity to IAV. *Boiled Lactobacillus plantarum 06CC2* increased the levels of antiviral effector molecules IFN-a, IFN-γ, IL-12, and the activity of NK cells at the early stage of infection (second day) (80). Oral administration of *Lactobacillus plantarum DK119* increased IL-12 and IFN-γ levels in BALF (81). *L. paracasei* increases the recruitment of dendritic cells in lung tissue after IAV infection (82). *Lactiplantibacillus plantarum 0111* oral pretreatment up-regulates ISG transcription 7 days after H9N2 infection while elevating CD3^+^CD4^+^TNF-α^+^T lymphocyte percentage and CD3^+^CD8^+^TNF-α^+^T lymphocyte percentage in the spleen and enhancing adaptive immunity to the virus (65).

In the late stages of infection, some probiotics can also inhibit inflammatory damage and promote tissue repair. *L. rhamnosus M21* reduces inflammatory damage in the lungs of IAV-infected mice and increases IFN-γ and IL-2 levels in lung lysates (85). *A. muciniphila* reduces the levels of pro-inflammatory factors IL-1β and IL-6 and increases the levels of inflammatory regulators IL-10, IFN-β, and IFN-γ in H7N9-infected mice (86). Combined treatment with the *probiotic L. mucosae 1025* and *B. breve CCFM1026* increases the level of butyrate in the cecal stool of IAV-infected mice and alleviates inflammatory infiltration in lung tissue (30). Some oral probiotics can act in both directions, creating an inflammatory environment conducive to viral clearance in the early stages of IAV infection and inhibiting excessive inflammatory activation in the later stages. Oral administration of *Bacteroides dorei* increased the expression of type 1 IFNs more rapidly in the early stages of infection (day 3) and reduced the viral load in the lungs, while in the later stages of infection (day 7) it reduced the levels of type 1 IFNs and other pro-inflammatory factors, which were beneficial for tissue repair (87). At the same time, *Bacteroides dorei* treatment also altered the composition of the GM, increasing *Bacteroides*, *Prevotella*, and *Lactobacillus* and decreasing *Escherichia*, *Shigella*, and *Parabacteroides (* 87). In addition to this, probiotic preparations can also benefit the effectiveness of influenza vaccines by promoting the production of IAV-specific antibodies. The dietary addition of prebiotic short-chain galacto-oligosaccharides and long-chain fructo-oligosaccharides increases influenza vaccine-specific T-helper 1 responses and specific B-cell activation in mLNs in mice, resulting in increased IgG1 and IgG2A levels (70).

In summary, probiotic preparations modulate the body’s antiviral immunity, including promoting the clearance of pathogens and repairing inflammatory damage in the later stages of IAV infection. However, different probiotics seem to play different roles and even have opposite effects, promoting inflammation or suppressing it. What we would like to achieve is an appropriate pro-inflammatory effect in the early stages of infection to promote pathogen clearance, and in the later stages to suppress inflammation to combat excessive inflammatory damage. This requires more in-depth research in the future to develop appropriate strategies for the use of probiotic preparations, such as using different combinations of probiotics at different periods of IAV infection.

### 6.2 Herbal medicine

The use of herbal medicine in the treatment of epidemic respiratory diseases has been used in China for more than 2,000 years. Existing research have shown that herbal treatment in influenza A has the effect of regulating GM, reducing viral load in the lungs, and alleviating inflammatory damage (**Table 1**). Unlike classical antiviral drugs, herbal remedies rely on multiple components and multiple therapeutic targets for their therapeutic effects. Our preliminary study used network pharmacology to analyze the active compounds in GeGen QinLian decoction and then screened the database to identify 89 influenza therapeutic-related targets (14). In addition to the direct therapeutic action of the drug molecules on their targets, herbal medicines also achieve therapeutic effects by regulating the composition of the GM. Transplantation of fecal microbiota isolated from mice orally administered GeGen QinLian Decoction to IAV-infected mice decreased the splenic Th17/Treg ratio and reduced the inflammatory damage effect in lung tissue, while an increase in *Akkermansia_muciniphila, Desulfovibrio_C21_c20*, and *Lactobacillus_salivarius* and a decrease in *Escherichia_coli* was observed in the intestine of the transplanted mice (14). Qin-Qiao-Xiao-Du decoction modulates the composition of the GM of IAV-infected mice and also inhibits virus proliferation in the lung and reduces plasma expression of IL-1α, IL-4, IL-12, and TNF-α (88). These effects may be related to the fact that it modulates the abundance of cyanoamino acid metabolism pathway-associated bacteria in the intestine, *Parabacteroides, Pediococcus*, and *Clostridium (* 88). Qingfeiyin decoction increased the abundance of *Coprococcus, Ruminococcus, Lactobacillus*, and *Prevotella* in the gut, reduced the abundance of *Escherichia, Parabacteroides, Butyricimonas*, and *Anacrotruncus* after H1N1 virus infection, down-regulated JAK-STAT, MAPK signaling pathway, reduced lung damage, reduced lung viral load, and improved survival rate (90). Cangma Huadu granules increased the abundance of *Bifidobacterium, Parasutterella, Bacteroides*, and *Faecalibaculum* in the intestine of mice at 7 days of PR8 infection, decreased the viral load and lung damage in the lungs, decreased TNF-a and IL-1β levels, and increased IL-10 levels (89). *Houttuynia cordata* and *Ephedra sinica* are both herbs commonly used in traditional Chinese medicine to treat influenza. Flavonoids and polysaccharides from *Houttuynia cordata*, reduced the proportion of pathogenic, *Vibrio* and *Bacillus* in the intestine and improved inflammatory damage in the intestinal and lung tissues of IAV mice (92–94). *Ephedra sinica* Stapf polysaccharide, the main component of *Ephedra sinica*, significantly increased the abundance of *Lactobacillales* and *Bifidobacteriaceae* in the intestine of mice 7 days after FM1 infection, and reduced IL-6, IL-8, and TNF-a in lung tissue (91).

**Table 1:** The role of herbal medicine in immune regulation of the gut- lung axis of influenza A.

Herbal medicine	Change of GM	Cytokines	Other Changes	
**Ge-Gen-Qin-** **Lian Decoction**	*Akkermansia_muciniphila*↑ *Desulfovibrio_C21_c20*↑ *Lactobacillus_salivarius*↑ *Escherichia_coli*↓	IL-6 ↓(serum) IL-17A ↓(serum) TGF-β ↓ (serum)	Th17/Treg↓(spleen), Th1/Th2↓(spleen) Claudin-1↑ (gut), ZO-1↑ (gut) Occludin ↑ (gut), NOD1↓ (gut) NOD2↓ (gut), RIP2↓ (gut) NF-κB↓ (gut), TLR7↓ (lung) MYD88↓ (lung), NF-κB p65↓ (lung)	(14)
**Qin-Qiao-Xiao-Du formula**	*Gemmiger*↑*, Anaerofustis*↑ *Adlercreutzia*↑, *Delftia*↓ *Streptococcus*↑, *Dehalobacteriu*↓ *Burkholderia*↓, *Prevotella*↓ *Butyrimimonas*↓	IL-1α ↓ (serum) IL-4 ↓ (serum) IL-12 ↓(serum) TNF-α ↓(serum)	Cyanoamino Acid Metabolism Pathway↑ (gut)	(88)
**Cangma Huadu granules**	*Bifidobacterium*↑, *Parasutterella*↑ *Bacteroides*↑ Faecalibaculum↑	IL-1β ↓(lung) TNF-α ↓ (lung) IL-10 ↑ (lung)	SOD↓ (lung), GSH-Px↓ (lung) MDA ↓(lung), Caspase-3↓ (lung) Bax/Bcl-2↓ (lung)	(89)
**Qingfeiyin Decoction**	*Coprococcus*↑, *Ruminococcus*↑ *Lactobacillus*↑, *Prevotella*↑ *Escherichia*↓, *Parabacteroides*↓ *Butyricimonas*↓, *Anacrotruncus*↓	NA	TRP channels↓ MAPK signaling pathway↓ TNFα signaling pathway↓ JAK-STAT signaling pathway↓	(90)
***Ephedra sinica Stapf polysaccharide***	*Lactobacillales*↑, *Listeriaceae*↓ *Moraxellaceae*↓, *Verru comicrobiaceae*↓ *Enterobacteriacea*↓ *Deferribacteraceae*↓ *Bifidobacteriaceae*↑, *Enterococcaceceaeae*↓ *Pepto*↓, *Clos-tridiales_vadinBB60_group*↓	TNF-α ↓(lung) IL-6 ↓(lung) IL-8 ↓(lung)	NA	(91)
***Houttuynia cordata polysaccharide***	*Vibrio↓, Acteroides↓* *Bacillus↓ Proteobacteria↓ Bf_Lachnoospiraceae↑*	IL-1β ↓(gut) IL-10 ↑(gut)	zonula occludens-1↑ (gut) ZO-1 ↑(gut) Th17/Treg↓ (GALT) TLR-4↓ (lung) NF-κB↓(lung) sIgA ↑(gut)	(92) (93) (94) (59)

In conclusion, the use of herbal medicines has the effect of modulating the GM after IAV infection, specifically by down-regulating the abundance of pathogenic bacteria and increasing probiotics. Modulation of the GM may be a key target for their ability to exert inhibition of IAV replication, promote immune clearance in the pre-infection period, and reduce inflammatory damage to tissues in the late infection period.

## 7 Summary and outlook

The intestinal tract is the largest immune system in the body, and GM has a powerful immunomodulatory function in the development of many immune-related diseases. In recent years, with the intensive study of the gut-lung axis, there has been an increasing interest in the role of GM among respiratory diseases, especially influenza A. However, fungi and viruses, as “dark matter” in the GM, have been neglected in past researches on the GM mechanisms of influenza A, as they are much less abundant than bacteria in the intestine. Moreover, viral nucleic acids lack a common sequence (95). However, with the development of sequencing technologies, more and more studies in recent years have also started to focus on the role of viruses and fungi in the gut, in other diseases. Clarifying the intestinal viral and fungal alterations caused by influenza will hopefully reveal the mechanism of the gut-lung axis of the body’s resistance to IAV infection, especially the intestinal virome. As influenza is a viral infection disease, there are many commonalities in the recognition and activation of the body’s immune system against IAV as well as intestinal viruses.

Current research has found that innate immunity to IAV, such as anti-viral processes mediated by ILCs, TLRs, RLRs, assembly of inflammasome, and subsequent adaptive immunity, including humoral immunity mediated by B cells and CD4^+^ helper T lymphocytes and cellular immunity mediated by CTL, is regulated by the GM and its metabolites. However, despite the fact that the intestine and lungs are two completely anatomically separated organs, most of the current studies have only described a parallel association between GM components and a specific IAV-associated immune-related molecule in the lungs. Therefore, it is worth pondering what mechanisms actually play a linking role between intestinal alterations and antiviral effects in the lungs? Certainty, blood and lymphatic circulation are important pathways of communication between the lung and intestine. Sine soluble GM metabolites can be transported to the lungs for function after entering the blood or lymphatic circulation. Among them, the role of SCFAs in influenza A is widely concerned by current research. SCFAs are recognized as positive substances in many inflammation-related diseases because of their anti-inflammatory effects (96, 97). Similarly, SCFAs play a tissue-protective role in influenza, preventing excessive tissue damage caused by the storm of inflammatory factors via promoting the accumulation of Treg cells at the local level of infection. However, it is also noteworthy that the antiviral mechanisms of SCFAs are complex and controversial. As we summarized, for example, acetate increases the production of IFN1s with antiviral effects through NLRP3-mediated activation of MAVS located on mitochondria, and, in addition, he restores pathogenic bacterial phagocytosis in macrophages after influenza infection. A high-fiber diet, an intervention that increases the production of intestinal SCFAs, contributes to the cytotoxic capacity of virus-specific CD8^+^ T lymphocytes. On the other hand, inflammatory suppression also means reduced clearance of pathogens, and some studies have found that high-fiber diets or supplementation with SCFAs early in viral infections increased viral loads and were not ameliorative, even for subsequent lung tissue damage, of intestinal inflammation (66, 98). It is likely that studies of the negative effects of SCFAs, a GM metabolite thought to have positive effects, will be abandoned for publication by researchers, and therefore more rigorous experimental designs are needed to elucidate the role of SCFAs in influenza. It might be more reasonable to evaluate the therapeutic role of SCFAs supplementation in influenza in different time periods (pathogen clearance and damage repair).

In addition, Tracking the migration of immune cells between the lung and intestine is expected to provide insight into the mechanisms by which the composition of the GM affects immunity in the gut-lung axis of IAV infection. Recent studies using immune cell labeling to demonstrate the migration of some migratory immune cells (NK, Th17, Treg, ILCs) between the lung and gut seem to provide us with good insight into the gut-lung axis. Th17 cells, a type of immune cell with strong tissue specificity (99).Chen Daofeng and his team found that CCL20, which is produced by lung tissue after IAV infection, can induce Th17 cells and Treg cells located in the intestine expressing CCR6 (the receptor for CCL20) to migrate to the lung-draining mLNs (59). Accordingly, are other circulating migratable immune cells involved in the gut-lung communication process during IAV infection? Do some probiotics, prebiotics, GM metabolites such as SCFAs, and herbal medicine is known to have antiviral efficacy remotely modulate anti-influenza immunity in the lung by regulating the migration of migratable immune cells?

**Figure 3:**
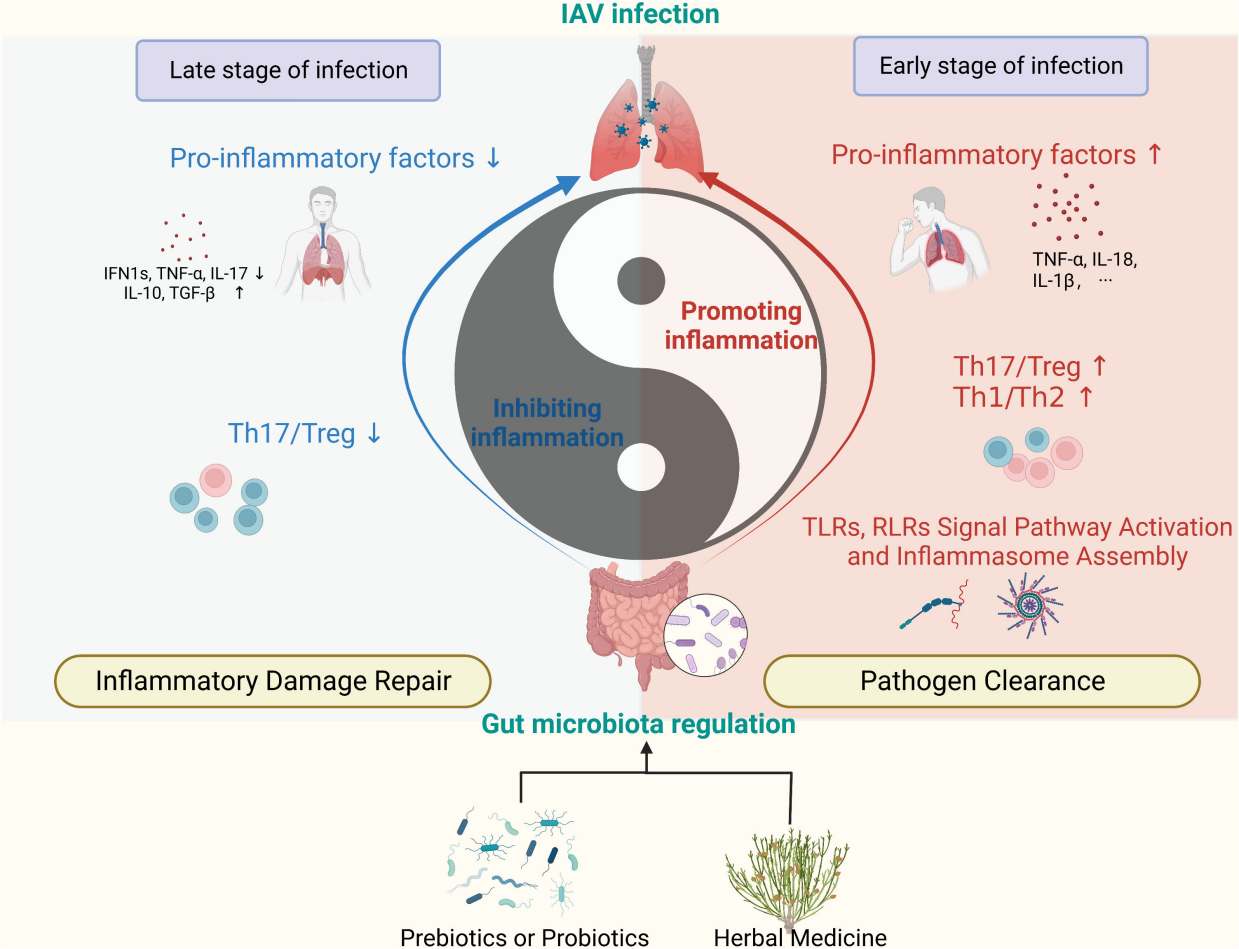
Regulation of GM promotes anti-IAV immune homeostasis. (Created with BioRender.com) The GM and its metabolites regulate immunity in the lungs in a bidirectional manner, manifesting as pro-inflammatory and anti-inflammatory effects. The use of prebiotics, probiotics, and herbal medicine can impact on the immune function of the lungs by modulating the components of the GM. (RLRs, RIG-I-like receptors; TLRs, Toll-like receptors).

In the pathophysiology of influenza, the body’s function of immune clearance of pathogens is analogous to yang, while the function of repairing tissues by suppressing excessive inflammatory responses is analogous to yin. It’s important for yin and yang to harmonize with each other (100). This allows for the rapid clearance of the IAV without causing serious pathological damage. Our research has found that regulation of gut components has the effect of balancing yin and yang (**Figure 3**). Specifically, regulation of the GM and its metabolites has a bidirectional regulatory role in the host’s anti-IAV immune response, enhancing the body’s inflammatory clearance of the virus in the early stages, a period of rapid pathogen replication, and conversely, serving to suppress inflammation and promote tissue repair in the later stages of infection. In conclusion, GM modulation therapy is a promising complementary therapy for the treatment of influenza A. A variety of probiotic applications have now been found to have anti-influenza effects, including reducing viral load in the lungs, improving survival, and reducing inflammatory damage to lung and intestinal tissues. However, an appropriate probiotic application strategy is still lacking. In addition, many herbal medicines have been found to exert antiviral effects in recent years and the mechanism of efficacy seems to be associated with the modulation of GM, but much research is still limited to the description of herbal-induced alterations in GM phenotypes. Fecal microbiota transplantation techniques, further specific pharmacodynamic flora isolation followed by implantation, or the application of germ-free mice could help further clarify the intestinal microecological mechanisms of herbal medicine for influenza A in the future.

## Author contributions

(I) Conception and design: HX, LD, and XC; (II) Fund financial support: LD, XC, and HX; (III) Manuscript writing: All authors; (IV) All authors contributed to the article and approved the submitted version.

## Glossary

**Table:**

ASC	apoptosis-associated speck-like protein containing a CARD
BIR	baculovirus inhibitor of apoptosis repeat domain
BALF	Bronchoalveolar Lavage Fluid
CARD	caspase recruitment domain
CTL	Cytotoxic T lymphocyte
DCs	dendritic cells
FM1	Influenza A virus (A/Fort Monmouth/1/1947 (H1N1))
GM	gut microbiota
GSDMD	gasdermin D
HA	Hemagglutinin
HDAC	histone deacetylase
IAV	Influenza A virus
iBALTs	inducible bronchus-associated lymphoid tissues
IFNAR	type I IFN receptor
IKK	NF-κB p65 inhibitor protein kinase
ILCs	Innate lymphoid cells
IRAK-4	IL-1R-associatedkinases 4
IRF3/7	IFN-regulatory factor 3/7
ISGs	interferon-stimulated genes
IκB	inhibitor of NF-κB
LPS	lipopolysaccharide
LRRs	leucine-rich repeats
MAVS	mitochondrial antiviral signaling
MHC II	major histocompatibility complex class II
mLNs	mediastinal lymph nodes
MyD88	myeloid differentiation primary response protein 88
NACHT	central nucleotide-binding and oligomerization domain
NF-κB	nuclear factor-κB
NLRP3	NOD-, LRR- and Pyrin Domain-Containing Protein 3
RLRs	RIG-I-like receptors
SFB	Segmented filamentous bacteria
ssRNA	single-stranded RNA
TAK	TGFβ-activated kinase
TANK1	TRAF associated NF-ĸB activator
Tfh	Follicular helper T cell
TLRs	Toll-like receptors
TRADD	TNFR associated death domain
TRAF3/6	TNF receptor-associated factor 3/6
TRAIL	Tumor necrosis factor-related apoptosis inducing ligand
PAMPs	pathogen-associated molecular patterns
PRRs	pathogen recognition receptors
PYD	pyrin domain
PR8	Influenza A virus (A/Puerto Rico/1934 (H1N1))
